# CDK5RAP3 suppresses Wnt/β-catenin signaling by inhibiting AKT phosphorylation in gastric cancer

**DOI:** 10.1186/s13046-018-0716-4

**Published:** 2018-03-14

**Authors:** Chao-Hui Zheng, Jia-Bin Wang, Man-Qiang Lin, Peng-Yang Zhang, Li-Chao Liu, Jian-Xian Lin, Jun Lu, Qi-Yue Chen, Long-Long Cao, Mi Lin, Ru-Hong Tu, Jian-Wei Xie, Ping Li, Chang-Ming Huang

**Affiliations:** 1 Department of Gastric Surgery, Fujian Medical University Union Hospital, Fuzhou, China; 2 Department of General Surgery, Fujian Medical University Union Hospital, Fuzhou, China; 3 Key Laboratory of Ministry of Education of Gastrointestinal Cancer, Fujian Medical University, Fuzhou, China; 4 Fujian Key Laboratory of Tumor Microbiology, Fujian Medical University, Fuzhou, China

**Keywords:** CDK5RAP3, AKT, GSK-3β, Gastric cancer, Survival

## Abstract

**Background:**

CDK5RAP3 was initially isolated as a binding protein of the CDK5 activator p35. Although CDK5RAP3 has been shown to negatively regulate the Wnt/β-catenin signaling pathway in gastric cancer by repressing GSK-3β phosphorylation, its in-depth mechanism has not been determined.

**Methods:**

Following CDK5RAP3 overexpression or knock down, CDK5RAP3 signaling pathways were investigated in gastric cancer cells by Western Blotting. Cell growth, invasion and migration were also evaluated in gastric cancer cell lines. We analyzed CDK5RAP3, AKT, p-AKT (Ser473), GSK-3β and p-GSK-3β (Ser9) expression in gastric tumor samples and adjacent non-tumor tissues from 295 patients using immunohistochemistry and Western Blotting. The prognostic significance of CDK5RAP3 and p-AKT (Ser473) was confirmed by a Log-rank test.

**Results:**

Our study demonstrated that the expression of p-AKT (Ser473) and p-GSK-3β (Ser9) was negatively correlated with CDK5RAP3 in stable gastric cancer cell lines. CDK5RAP3 repressed AKT phosphorylation, which promoted GSK-3β phosphorylation, thereby suppressing β-catenin protein expression and, consequently, gastric cancer. The protein level of CDK5RAP3 was markedly decreased in most gastric tumor tissues compared with adjacent non-tumor tissues, and the levels of p-AKT (Ser473) and p-GSK-3β (Ser9) were also negatively correlated with those of CDK5RAP3. The prognostic value of CDK5RAP3 for overall survival was found to be dependent on AKT phosphorylation.

**Conclusion:**

Our results demonstrated that CDK5RAP3 negatively regulates the Wnt/β-catenin signaling pathway by repressing AKT phosphorylation, which leads to better survival of patients with gastric cancer.

## Background

Gastric cancer is the second leading cause of cancer death, with East Asia accounting for more than half of the annual cases [1]. It is of great importance to identify new molecular markers that may help evaluate the prognosis or develop novel therapies for gastric cancer. Cyclin dependent kinase 5 (CDK5) regulatory subunit-associated protein 3 (CDK5RAP3, also called C53/LZAP) was originally identified as a binding protein of Cdk5 activator p35 by the yeast two-hybrid assay [2]. Our previous study demonstrated that CDK5RAP3 expression was downregulated in gastric cancer, which correlated with poor prognosis, and that CDK5RAP3 could inhibit Wnt/β-catenin signaling by blocking GSK-3β phosphorylation at Ser9 [3]. However, it remains unclear how CDK5RAP3 suppresses GSK-3β phosphorylation. GSK-3β phosphorylation is mediated by several kinases, including AKT, protein kinase A (PKA) and protein kinase C (PKC), and phosphorylation of serine 9 has been shown to suppress GSK-3β activity [4, 5].

AKT (protein kinase B) is a serine/threonine kinase that was originally discovered as an oncogene transduced by an acute transforming retrovirus isolated from an AKR thymoma [6]. Numerous studies have shown that AKT is a critical signaling node within all cells of higher eukaryotes and one of the most important and versatile protein kinases at the core of human physiology and disease [7, 8]. Activation of AKT has frequently been reported in many human cancers, including carcinomas of the breast, lung, pancreas and thyroid, as well as in gastric cancer [9]. In addition, this kinase appears to play an important role in cancer development, progression, and therapeutic resistance.

In gastric cancer, activated AKT induces the in-activation of GSK-3β, thereby activating Wnt/β-catenin signaling [10]. There have been many breakthroughs regarding the upstream regulatory mechanisms of AKT; however, whether AKT/GSK-3β/β-catenin signaling is regulated by CDK5RAP3 in gastric cancer has not been explored. One possibility may be that CDK5RAP3 could bind with AKT at GSK-3β binding sites and physically isolate GSK-3β.

In this study, we found that CDK5RAP3 blocked AKT phosphorylation to inhibit β-catenin signaling, which promoted gastric cancer cell proliferation, invasion and migration. We also investigated the prognostic value of CDK5RAP3 in gastric cancer and showed that it depended on AKT activity.

## Methods

### Human gastric tumor tissues

Human gastric tumor tissues with detailed clinic pathologic parameters were obtained from 295 patients at Fujian Medical University Union Hospital (Fujian, China). All patients underwent radical gastrectomy from 2010 to 2015. None of the patients underwent preoperative chemotherapy or radiotherapy. Postoperative adjuvant chemotherapy was performed with 5-fluorouracil-based drugs plus oxaliplatin in advanced cases. The pathologic stage of the tumor was reassessed according to the 2010 International Union Against Cancer (UICC) TNM classification of gastric cancer (seventh edition) [11]. Adjacent non-tumor tissues were obtained from an area at least 5 cm away from the gastric tumor. All fresh specimens were stored in liquid nitrogen after resection until protein or RNA extraction. The 209 paraffin-embedded gastric tumor and adjacent non-tumor tissues were collected for immunohistochemistry (IHC) from 2010 to 2014. The 86 fresh gastric tumor tissues and adjacent non-tumor tissues were subjected to western blotting between 2014 and 2015. This study was approved by the ethics committee of Fujian Medical University Union Hospital, and written consent was obtained from all involved patients.

### Follow-up

All patients were systematically followed up by trained doctors following the institutional follow-up protocol via several approaches, including outpatient services, letters, telephone, mail or visits. Follow-up was conducted every 3 months during the first year and every 6 months after the second year, and all surviving patients were followed for more than three years. Survival time was defined as the time from the date of surgery until the last follow-up or the date of death. All 209 patients involved in the IHC analysis completed the follow-up.

### Tissue microarray (TMA)

A series of TMAs containing gastric cancer samples were constructed. Briefly, all the gastric cancer tissues were reviewed by a pathologist, and representative areas free from necrotic and hemorrhagic materials were premarked in the paraffin blocks. For each sample, a 1.5-mm core was punched from the donor blocks and transferred to the recipient paraffin block at defined array positions using a tissue microarray instrument. Several serial sections (4 μm in thickness) were cut from all TMAs, and one section from each TMA was stained with hematoxylin-eosin and served as a reference.

### Immunohistochemistry (IHC) and scoring

Paraffin blocks that contained sufficient formalin-fixed tumor specimens were serially sectioned at 4 μm and mounted on silane-coated slides for IHC analysis. The sections were deparaffinized with dimethylbenzene and rehydrated through an ethanol gradient, including 100, 95, 85 and 75% ethanol. Antigen retrieval was performed with 0.01 mol/L sodium citrate buffer (pH 6.0) in an autoclave at 121 °C for 2 min, and endogenous peroxidase was blocked by incubation with 3% hydrogen peroxide for 10 min at room temperature. The slides were then washed in phosphate-buffered saline (PBS), blocked with 10% goat serum (ZhongShan Biotechnology, China) for 30 min and incubated with antibodies in a humidified chamber at 4 °C overnight. Following three washes in PBS, the sections were incubated with HRP-conjugated secondary antibody for 30 min at room temperature. Next, the signal was developed with diaminobenzidine (DAB) solution, and all the slides were counterstained with 20% hematoxylin. Last, the slides were dehydrated and mounted with cover slips. For negative controls, the primary antibody diluent was used instead of the primary antibody. The staining intensity was scored as 0 to 3. The heterogeneity of staining was scored as 0 to 3, depending on the percentage of tumor cells that were positively stained (3). To obtain an IHC score that considers the IHC signal intensity and the frequency of positive cells, we generated a composite expression score (CES) ranging from 0 to 9. A CES of 0, 1, 2 and 3 was defined as low expression, but a CES of 4, 6 and 9 was defined as high expression.

### Western blotting analysis

The methods were described as previously described [3]. Anti-GSK-3β (#9832), AKT (pan) (#4685), phosphorylated AKT (Ser473) (#4060), GSK-3β (Ser9) (#12456) and phosphorylated GSK-3β (Ser9) (#5558) were purchased from Cell Signaling Technology. Anti-CDK5RAP3 (ab157203), E-cadherin (ab1416), N-cadherin (ab76057), Vimentin (ab8978), and GAPDH (ab181602) antibodies were purchased from Abcam. AKT siRNA (#6211) and control siRNA (#6568) were purchased from Cell Signaling Technology. We corrected the loading error based on loading controls and compared the expression levels of target proteins in gastric tumor and adjacent non-tumor tissues. The protein expression in tumors was defined as high level when it was higher than that in normal tissue but was defined as low level when it was lower than that in normal tissue.

### Cell culture

Human gastric cancer cell lines AGS and HGC-27 were obtained from the Cell Line Bank at the Chinese Academy of Sciences. All the cell lines were confirmed to be free of mycoplasma contamination by PCR and culture. The species origin was confirmed with PCR. The identity of the cell line was authenticated with short tandem repeat (STR) profiling. These cell lines were cultured in RPMI 1640 (Gibco, Grand Island, NY) supplemented with 10% fetal bovine serum (FBS) (Gibco, Grand Island, NY) and incubated at 37 °C in a humidified atmosphere containing 5% CO_2_.

### Establishment of cell lines

The cell lines were established as previously described [3].

### Cell proliferation

Cell proliferation was assessed using the CCK8 assay and colony formation assay. Details of cell proliferation are described in the Additional file 1.

### Cell migration and invasion assays

Cell migration and invasion assays were assessed in Transwell chambers (polycarbonate filters of 8-μm porosity, BD Bioscience) and by the wound healing assay. The details are described in the Additional file 1.

### Statistical analysis

All statistical analyses were performed using SPSS 18.0 for Windows (SPSS, Chicago, IL) and Prism 5.0 software (GraphPad). The chi-squared test was used to evaluate the difference in proportions, and Student’s t-tests were used to evaluate continuous variables. Overall survival curves were calculated with the Kaplan-Meier method; the log-rank test was used to detect differences between groups. Independent prognostic factors were identified with a Cox proportional hazards regression model. *P* < 0.05 was considered statistically significant, and all *P* values were two-sided.

## Results

### CDK5RAP3 controls β-catenin protein expression via AKT to regulate GSK-3β phosphorylation

**Fig. 1:**
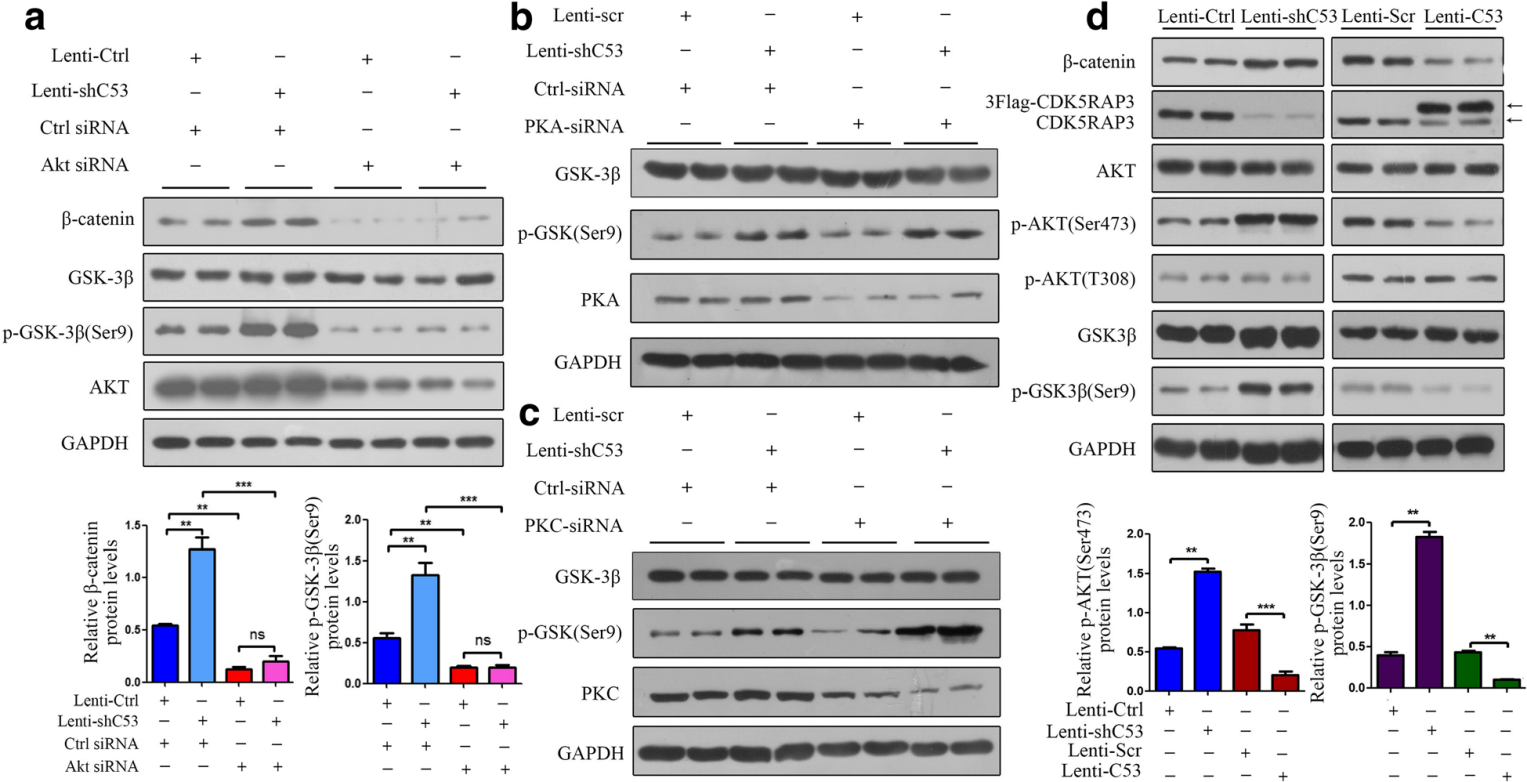
CDK5RAP3 controls β-catenin protein expression via AKT to regulate GSK-3β phosphorylation. **a** The Lenti-ctrl- and Lenti-shC53-treated AGS cells were transfected with AKT siRNA. The effects of AKT knockdown were confirmed by Western Blotting. **b**-**c**) Knockdown of PKA and PKC had no effect on p-GSK3β (S9) expression. **d** AGS cells in which CDK5RAP3 was stably overexpressed or knocked down were generated. CDK5RAP3 expression changes were confirmed by Western Blotting. Knockdown of CDK5RAP3 increased the protein levels of p-AKT (S473) and p-GSK3β (S9) in HGC-27 cells. Overexpression of CDK5RAP3 resulted in the opposite effects

Our previous study had confirmed that CKD5RAP3 suppressed tumor cells through the inhibition of β-catenin signaling by regulating GSK-3β phosphorylation. To study the interactions among AKT, CDK5RAP3 and GSK-3β, CDK5RAP3 was stably overexpressed or knocked down in AGS cells. CDK5RAP3 expression was confirmed using Western Blotting, and the effect of AKT on CDK5RAP3 function in gastric cancer cells was assessed using AKT siRNA. In CDK5RAP3-overexpressing AGS cells, AKT siRNA reduced AKT levels and also reduced phosphorylated GSK-3β (Ser9) and β-catenin levels, even in the presence of Lenti-shC53 (Fig. 1a). However, PKA and PKC silencing did not affect GSK-3β phosphorylation (Ser9) (Fig. 1b-c), which meant that CDK5RAP3 controlled GSK-3β phosphorylation without phosphorylating PKA and PKC. In addition, knockdown of CDK5RAP3 in gastric cancer cells led to upregulated AKT phosphorylation at Ser473, GSK-3β phosphorylation at Ser9 and β-catenin expression, whereas overexpression of CDK5RAP3 led to the opposite effects (Fig. 1d), indicating that CDK5RAP3 may regulate β-catenin activity by suppressing AKT phosphorylation at Ser473.

### CDK5RAP3 exerted tumor suppressive functions by blocking AKT activation

**Fig. 2:**
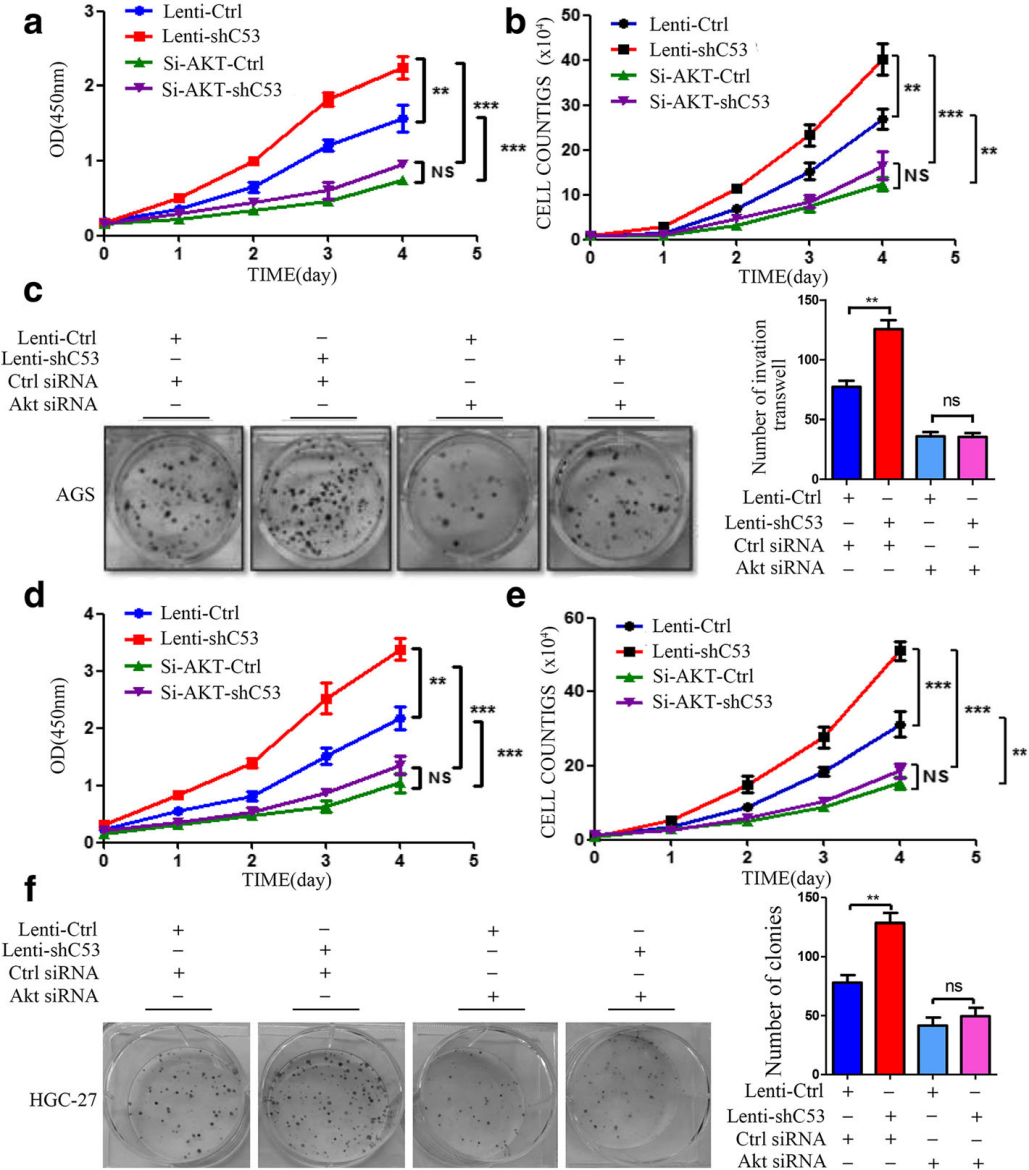
AKT silencing repressed the tumorigenicity of CDK5RAP3 knockdown gastric cancer cells. **a**-**b**, **d**-**e**) The stimulatory effect of CDK5RAP3 downregulation on AGS and HGC-27 cell proliferation was rescued by AKT siRNA transfection, as shown by both the CCK8 proliferation assay (**a**, **d**) and cell counting assay (**b**, **e**) (**, *P* < 0.01; ***, *P* < 0.001; ns, no significance). (**c**, **f**) The stimulatory effect of CDK5RAP3 downregulation on AGS and HGC-27 cell colony formation was rescued by AKT siRNA transfection. The data are presented as the mean ± SD (**, *P* < 0.05; ns, no significance)

**Fig. 3:**
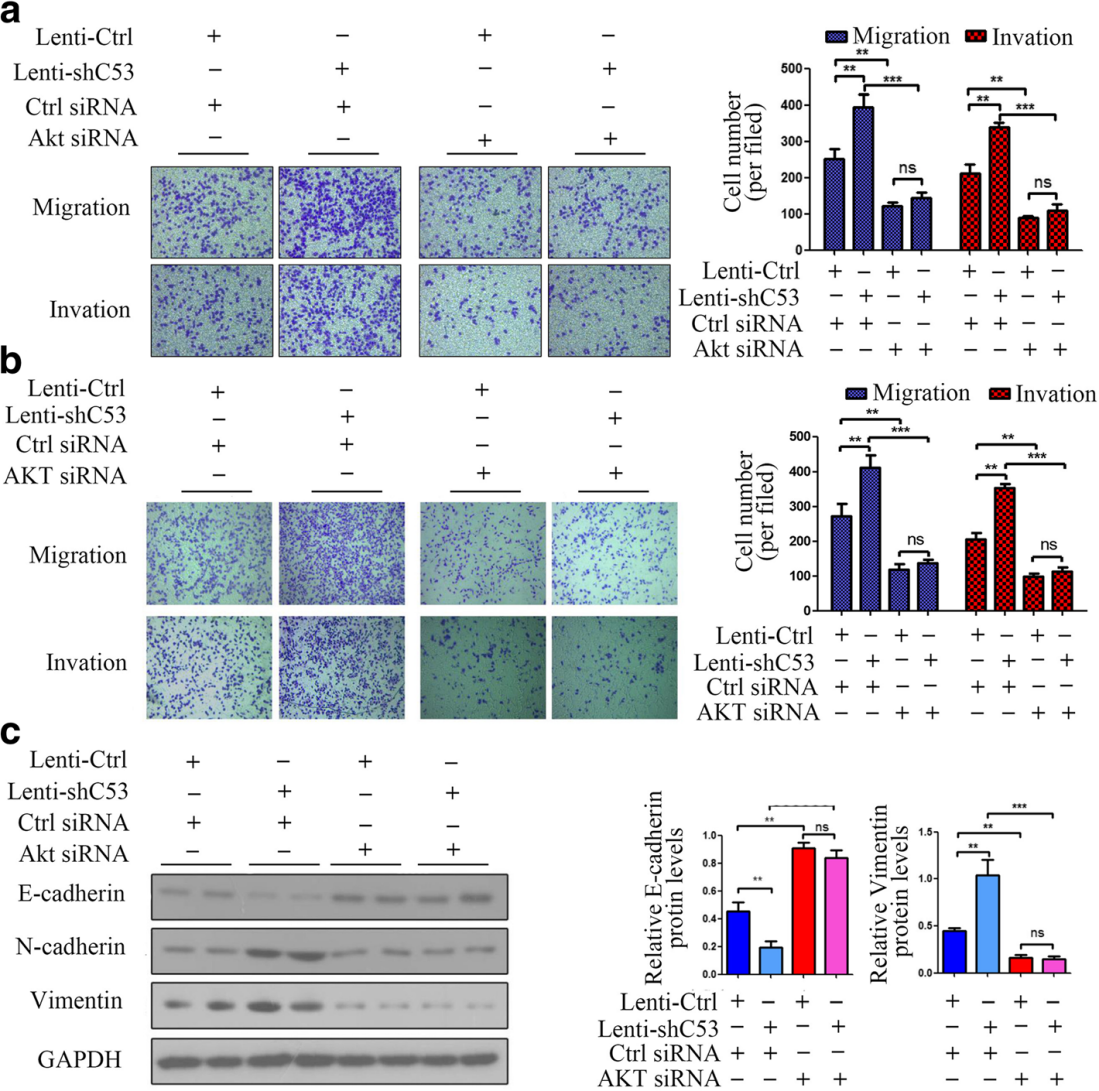
AKT silencing repressed the migration and invasion of CDK5RAP3 knockdown gastric cancer cells. **a**-**b** The stimulatory effect of CDK5RAP3 downregulation on AGS and HGC-27 cell migration and invasion was rescued by AKT siRNA transfection. The data are presented as the mean ± SD (**, *P* < 0.01; ***, *P* < 0.001; ns, no significance). **c** When AKT was knocked down, the protein levels of E-cadherin, N-cadherin and Vimentin were not significantly changed whether CDK5RAP3 was knocked down or overexpressed (**, *P* < 0.01; ***, *P* < 0.001; ns, no significance)

Inhibition of AKT in CDK5RAP3 stably knockdown AGS and HGC-27 cells abrogated accelerated proliferation (Fig. 2a-b, d-e), colony formation (Fig. 2c, f), and cell migration and invasion (Fig. 3a-b). We also investigated the role of AKT in the CDK5RAP3-induced epithelial–mesenchymal transition (EMT) process. When AKT was knocked down, the protein levels of EMT markers (E-cadherin, N-cadherin and Vimentin) were not significantly changed whether CDK5RAP3 was knocked down or overexpressed (Fig. 3c). These results indicate that the regulation of CDK5RAP3 on EMT depends on the activity of AKT in gastric cancer.

### AKT and GSK-3β are involved in CDK5RAP3 signaling in gastric cancer

**Fig. 4:**
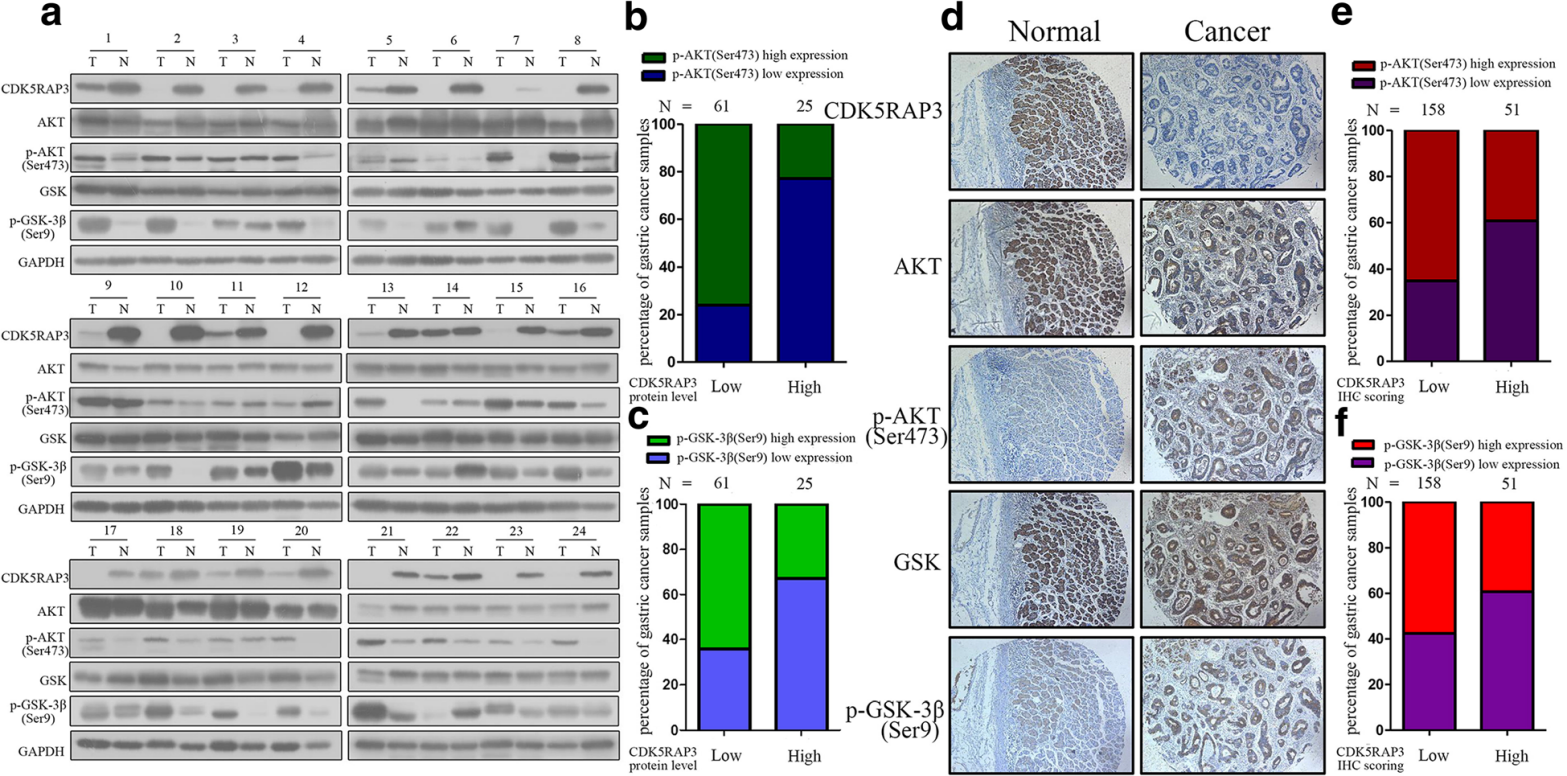
The clinical value of CDK5RAP3 depends on AKT activity. **a** The protein levels of CDK5RAP3/AKT/p-AKT (S473)/GSK3β/p-GSK3β (S9) in gastric tumor tissues and adjacent non-tumor tissues from 86 patients were measured by western blotting. The representative results are shown. **b** There was an inverse correlation between CDK5RAP3 protein level and p-AKT (S473) expression in gastric tumor tissues. **c** There was an inverse correlation between CDK5RAP3 protein level and p-GSK3β (S9) expression in gastric tumor tissues. **d** The expression of CDK5RAP3, GSK, p-GSK3β (S9), AKT, p-AKT (S473) protein in gastric tumor tissues and adjacent non-tumor tissues was analyzed using IHC (representative results are shown). **e** There was an inverse correlation between CDK5RAP3 IHC scoring and p-AKT (S473) expression in gastric tumor tissues. **f** There was an inverse correlation between CDK5RAP3 protein level and p-GSK3β (S9) expression in gastric tumor tissues

CDK5RAP3, p-AKT (Ser473) and p-GSK-3β (Ser9) protein levels were detected in gastric tumor tissues and adjacent non-tumor tissues from an additional 86 patients using Western Blotting. The 24 representative samples were shown in Fig. 4a. In cancer samples with low CDK5RAP3 protein levels, the proportion of samples exhibiting p-AKT (Ser473) expression was significantly higher than that exhibiting high CDK5RAP3 expression (77.0% vs. 24.0%) (Fig. 4b). In addition, in cancer samples with low CDK5RAP3 protein levels, the proportion of samples exhibiting p-GSK-3β (Ser9) expression was also significantly higher than that exhibiting high CDK5RAP3 expression (64.0% vs. 32.0%) (Fig. 4c).

To further verify the associations among CDK5RAP3, p-GSK-3β (Ser9) and p-AKT (Ser473), we detected the expression of CDK5RAP3, p-GSK-3β (Ser9) and p-AKT (Ser473) in the 209 gastric cancer samples using IHC. In cancer samples with low CDK5RAP3 expression, the staining of phosphorylated AKT (S473) was significantly darker than in those with high CDK5RAP3 expression; this was also observed regarding phosphorylated GSK-3β (Ser9) (Fig. 4d). In low CDK5RAP3 IHC scoring samples, phosphorylated AKT (S473) protein expression was significantly higher than that in high CDK5RAP3 IHC scoring samples (65.2% vs. 39.2%) (Fig. 4e). The trend of phosphorylated GSK-3β (Ser9) was the same (57.6% vs. 39.2%) (Fig. 4f). These results suggest that CDK5RAP3 regulates tumor cells by regulating AKT phosphorylation and GSK-3β phosphorylation.

### The prognostic value of CDK5RAP3 in gastric cancer depends on p-AKT (Ser473) expression

To evaluate the prognostic value of combined CDK5RAP3 and p-AKT (Ser473) expression, the Cox proportional hazards regression model was used (Table 1). Univariate analysis revealed that tumor size, histology, depth of invasion, lymph node metastasis, distant metastasis and CDK5RAP3 and p-AKT (Ser473) expression were associated with overall survival. Multivariate Cox regression analyses showed that T, N and M stages as well as CDK5RAP3 and p-AKT (Ser473) expression remained independent prognostic factors.

**Table 1:** Analysis of the Correlation Between Clinicopathological Parameters and Survival of Patients

		Univariate analysis		Multivariate analysis		
	HR	95% CI	*P* value	HR	95% CI	*P* value
Age (years)						
< 65 vs. ≥ 65	0.94	0.55–1.59	0.809			
Gender						
Male vs. Female	0.73	0.36–1.48	0.377			
Tumor size (mm)						
< 50 mm vs. ≥50 mm	1.58	1.13–2.71	0.043	0.861	0.48–1.55	0.616
Histology						
Well/Moderately vs. Poor	1.59	1.22–2.72	0.045	1.129	0.64–1.98	0.674
Tumor location						
Upper vs. Middle vs. Low vs. ≥2 regions	0.84	0.64–1.11	0.219			
Depth of invasion						
pT1 vs. pT2 vs. pT3 vs. pT4	3.47	2.01–5.97	<0.001	2.57	1.50–4.40	0.001
Lymph node metastasis						
pN0 vs. pN1 vs. pN2 vs. pN3	2.59	1.74–3.85	<0.001	2.16	1.38–3.38	0.001
Distant metastasis						
pM0 vs. pM1	6.94	3.42–14.07	<0.001	3.34	1.54–7.23	0.002
CDK5RAP3 expression						
Low vs. High	0.84	0.72–0.97	0.014	0.89	0.78–0.98	0.035
p-AKT(Ser473) expression						
Low vs. High	4.17	2.10–8.28	<0.001	3.99	1.97–8.08	<0.001

**Fig. 5:**
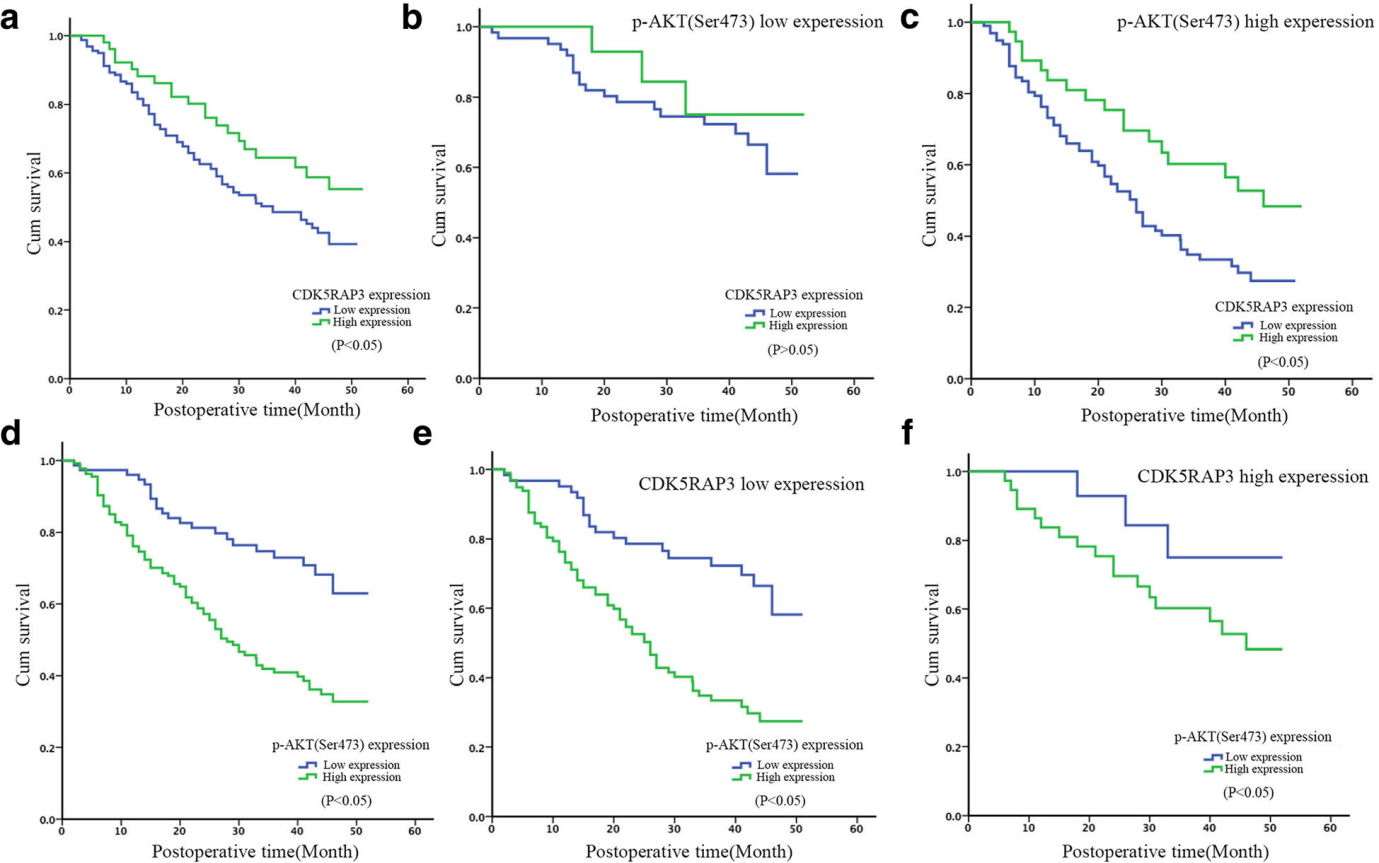
The prognostic value of CDK5RAP3 is dependent on p-AKT (Ser473) expression. **a** Kaplan-Meier survival curve of gastric cancer patients with low or high CDK5RAP3 expression (*P* < 0.05, log-rank test). **b** Kaplan-Meier survival curve of patients with high p-AKT (Ser473) expression and low or high CDK5RAP3 expression (*P* < 0.05, log-rank test). **c** Kaplan-Meier survival curve of patients with low p-AKT (Ser473) expression and low or high CDK5RAP3 expression (*P* > 0.05, log-rank test). **d**-**f** Kaplan-Meier survival curves of patients with low or high p-AKT (Ser473) expression were not significantly affected by CDK5RAP3 expression (*P* < 0.05, log-rank test)

Next, we compared the overall survival of patients categorized by CDK5RAP3 and p-AKT (Ser473) expression. The overall survival rate of patients with low CDK5RAP3 expression was significantly lower than that of patients with high CDK5RAP3 expression (Fig. 5a). When p-AKT (S473) expression was low, a significant difference in overall survival was not observed between patients with low or high CDK5RAP3 expression (Fig. 5b). However, when p-AKT (Ser473) expression was high, gastric cancer patients with low CDK5RAP3 expression had a poorer prognosis than those with high CDK5RAP3 expression (*P* < 0.05, Fig. 5c). On the other hand, CDK5RAP3 expression did not affect the prognostic role of p-AKT (S473) expression (Fig. 5e-g). These results confirm that the clinical value of CDK5RAP3 is dependent on AKT activity.

## Discussion

CDK5RAP3 is an evolutionally conserved protein that is closely related to the occurrence and development of various cancers. CDK5RAP3 has been shown to function as a tumor suppressor in head and neck squamous cell carcinomas (HNSCCs), and forced expression of CDK5RAP3 suppresses NF-kB activity [12]. In addition, low expression of CDK5RAP3 inhibits the invasion, migration and proliferation of U2OS osteosarcoma cells [13]. However, other reports have demonstrated the opposite, suggesting that the function of CDK5RAP3 may be tissue specific. For example, Mak et al. discovered that CDK5RAP3 was widely overexpressed in hepatocellular carcinoma (HCC) and promoted HCC metastasis through p21-induced protein kinase4 (PAK4) activation [14]. CDK5RAP3 was also shown to stimulate vascular endothelial cell proliferation [15]. In addition, Chen et al. illustrated that CDK5RAP3 expression positively correlated with the grade and depth of invasion in colon adenocarcinoma [16]. The activities of CDK5RAP3 vary in different cancer types, reflecting the importance of CDK5RAP3 in tumorigenesis and cancer metastasis, and the specific function of CDK5RAP3 remains unknown.

Previously, we have investigated the role of CDK5RAP3 in gastric cancer [3]. Our study demonstrated that CDK5RAP3 expression was markedly decreased in gastric tumor tissues and led to significantly reduced cancer cell proliferation, migration and invasion and tumor xenograft growth by inhibiting β-catenin. Importantly, we found that CDK5RAP3 was a significant regulator of GSK3β phosphorylation in gastric cancer, leading to the subsequent degradation of β-catenin and influencing the prognosis of gastric cancer patients. Several studies have also reported that CDK5RAP3 is implicated in the regulation of protein phosphorylation. In HNSCCs, CDK5RAP3 was shown to suppress NF-κB by reducing the phosphorylation of RelA [12]. Lin KY et al. found that CDK5RAP3 also suppressed Wnt/β-catenin signaling via the suppression of GSK-3β phosphorylation in zebrafish embryos [17]. However, how CDK5RAP3 suppresses GSK-3β phosphorylation is not well understood. Therefore, the current study sought to elucidate the mechanisms underlying the CDK5RAP3-mediated regulation of GSK-3β phosphorylation.

GSK-3β, which is involved in the formation of various tumors, functions as a critical downstream regulatory switch for numerous signaling pathways, including cellular responses to Wnt/β-catenin [18, 19]. In the Wnt/β-catenin signaling pathway, activated AKT binds to the axin-GSK3β complex in the presence of Dishevelled (Dvl), phosphorylating GSK-3β at Ser9 and increasing the levels of free β-catenin [20]. AKT activation is dependent on its phosphorylation at T308 and S473 [21, 22]. At the membrane, AKT is phosphorylated at T308 by phosphoinositide-dependent kinase-1 (PDK-1) and becomes partially activated. Additional activation at S473 by a still-undefined kinase results in fully activated AKT. Activated AKT translocates to the cytosol and nucleus to phosphorylate its substrates [23–25].

In this study, knockdown of AKT reversed the effect of CDK5RAP3 downregulation, whereas PKA and PKC had no effect, implicating that AKT, rather than PKA or PKC, mediates the tumor suppressor role of CDK5RAP3 in gastric cancer. Furthermore, CDK5RAP3 expression is frequently found to be dysregulated in gastric cancer at the protein level, while p-AKT (S473) and p-GSK-3β (Ser9) expression is upregulated. Significantly, AKT activation affects the prognostic value of CDK5RAP3 for overall survival, whereas CDK5RAP3 expression does not affect the prognostic value of AKT, which provides clinical support for the tumor suppression mechanism of CDK5RAP3.

## Conclusion

**Fig. 6:**
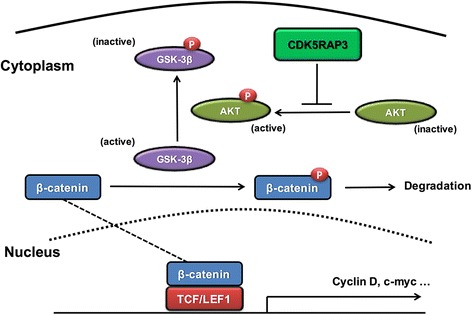
A schematic model for the CDK5RAP3 - AKT - GSK3β - β-catenin pathway in gastric cancer. CDK5RAP3 suppresses the inhibitory phosphorylation of GSK3β via repressing AKT activation, enabling active GSK3β phosphorylate β-catenin for its degradation in gastric cancer

In summary, our results clarified that CDK5RAP3 blocks AKT phosphorylation to inhibit β-catenin signaling (Fig. 6), thereby influencing the biological behavior and prognosis of gastric cancer, which supplements the mechanism described in our previous study.
